# Supplementary material for: Using supermarket loyalty card data to investigate seasonal variation in laxative purchases in the UK
Source: PLOS Digit Health. 2026 Mar 18;5(3):e0000963. doi: 10.1371/journal.pdig.0000963 (PMC12998798; doi:10.1371/journal.pdig.0000963)
Supplement: S1 Text — (DOCX) [file pdig.0000963.s001.docx]

**S1 Laxative Product Code Book**

**S1 Table A:** Code book for all laxative products in the retailer dataset, including maximum dosage and source.

| **Product** | **Active Ingredient** | **Type** | **Quantity (tablets)** | **Quantity (ml)** | **Maximum Daily Dose (tablets or ml)** | **Max Dose Duration** | **Source** |
| --- | --- | --- | --- | --- | --- | --- | --- |
| LIQ PARAFF 150ML(P) | Paraffin | Non-stimulant | - | 150 | 30 | 5 | 2 – 6 x 5ml spoonful’s <https://www.mypharmacy.co.uk/product/liquid-paraffin-150ml/> |
| LIQ PARAFF 200ML (P) | Paraffin | Non-stimulant | - | 200 | 30 | 6.67 | 2 – 6 x 5ml spoonful’s <https://www.mypharmacy.co.uk/product/liquid-paraffin-150ml/> |
| FYBOGEL HIFI OR 30 (G) | Ispaghula husk | Non-stimulant | 30 |  | 2 | 15 | One sachet morning and evening <https://www.senokot.co.uk/our-products/fybogel-hi-fibre-sachets/> |
| FYBOGEL HI FIB ORAN 10 (E) (G) | Ispaghula husk | Non-stimulant | 10 |  | 2 | 5 | One sachet morning and evening <https://www.senokot.co.uk/our-products/fybogel-hi-fibre-sachets/> |
| FYBOGE HI FBRLEM 10 (E) (G) | Ispaghula husk | Non-stimulant | 10 |  | 2 | 5 | One sachet morning and evening <https://www.senokot.co.uk/our-products/fybogel-hi-fibre-sachets/> |
| ROI FYBOGEL ORANGE 10 (P) | Ispaghula husk | Non-stimulant | 10 |  | 2 | 5 | One sachet morning and evening <https://www.senokot.co.uk/our-products/fybogel-hi-fibre-sachets/> |
| Fybogel lemon sachets 60s | Ispaghula husk | Non-stimulant | 60 |  | 2 | 30 | One sachet morning and evening <https://www.senokot.co.uk/our-products/fybogel-hi-fibre-sachets/> |
| Fybolgel granules sachet 30s | Ispaghula husk | Non-stimulant | 30 |  | 2 | 15 | One sachet morning and evening <https://www.senokot.co.uk/our-products/fybogel-hi-fibre-sachets/> |
| LACTULOSE 200ML P | Lactulose | Non-stimulant |  | 200 | 45 | 4.44 | The starting dose is 15-45ml per day <https://www.medicines.org.uk/emc/files/pil.5525.pdf> |
| Lactulose Solution 500ml | Lactulose | Non-stimulant |  | 500 | 45 | 11.11 | The starting dose is 15-45ml per day <https://www.medicines.org.uk/emc/files/pil.5525.pdf> |
| Lactulose 300ml (P) | Lactulose | Non-stimulant |  | 300 | 45 | 6.67 | The starting dose is 15-45ml per day <https://www.medicines.org.uk/emc/files/pil.5525.pdf> |
| Lactulose Sachets 10s (P) | Lactulose | Non-stimulant | 10 |  | 1 | 10 | The dose may be given once daily <https://www.chemist-4-u.com/lactulose-10g-15ml-oral-solution-10-sachets-brand-may-vary> |
| LACTULOSE (P) | Lactulose | Non-stimulant |  | 300 | 45 | 6.67 | The starting dose is 15-45ml per day <https://www.medicines.org.uk/emc/files/pil.5525.pdf> |
| LACTULOSE 500ML B | Lactulose | Non-stimulant |  | 500 | 45 | 11.11 | The starting dose is 15-45ml per day <https://www.medicines.org.uk/emc/files/pil.5525.pdf> |
| ROI Laxose solution 500ml | Lactulose | Non-stimulant |  | 500 | 45 | 11.11 | The starting dose is 15-45ml per day <https://www.medicines.org.uk/emc/files/pil.5525.pdf> |
| Lactulose solution 500ml (P) | Lactulose | Non-stimulant |  | 500 | 45 | 11.11 | The starting dose is 15-45ml per day <https://www.medicines.org.uk/emc/files/pil.5525.pdf> |
| Lactulose solution 300ml (P) | Lactulose | Non-stimulant |  | 300 | 45 | 6.67 | The starting dose is 15-45ml per day <https://www.medicines.org.uk/emc/files/pil.5525.pdf> |
| ROI Laxaclear sachets 5.9g 10s (P) | Lactulose | Non-stimulant | 10 |  | 1 | 10 | The dose may be given once daily <https://bnf.nice.org.uk/drugs/lactulose/#indications-and-dose> |
| LACTULOSE 300ML | Lactulose | Non-stimulant |  | 300 | 45 | 6.67 | The starting dose is 15-45ml per day <https://www.medicines.org.uk/emc/files/pil.5525.pdf> |
| LACTULOSE 500ML | Lactulose | Non-stimulant |  | 500 | 45 | 11.11 | The starting dose is 15-45ml per day <https://www.medicines.org.uk/emc/files/pil.5525.pdf> |
| ROI LAXOSE 300ML (P) | Lactulose | Non-stimulant |  | 300 | 45 | 6.67 | The starting dose is 15-45ml per day <https://www.medicines.org.uk/emc/files/pil.5525.pdf> |
| K LACTULOSE SYR 500ML | Lactulose | Non-stimulant |  | 500 | 45 | 11.11 | The starting dose is 15-45ml per day <https://www.medicines.org.uk/emc/files/pil.5525.pdf> |
| ZZNOVA LACTLSE 200ML (P) | Lactulose | Non-stimulant |  | 200 | 45 | 4.44 | The starting dose is 15-45ml per day <https://www.medicines.org.uk/emc/files/pil.5525.pdf> |
| NORGALAX MICRO-ENEMAS 6 | Docusate sodium | Non-stimulant | 6 |  | 2 | 3 | You will only need to use one tube, but if needed, you can use a second later on in the day. <https://www.chemist-4-u.com/norgalax-disposable-microenema-10g-6-pack> |
| NORMACOL SCHT 60 | Sterculia | Non-stimulant | 60 |  | 4 | 15 | 1-2 sachets, once or twice daily after meals <https://www.chemistcounterdirect.co.uk/normacol-sachets-7g-60.html> |
| NORMACOL GRANS 500G | Sterculia | Non-stimulant | 60 |  | 4 | 15 | 1-2 sachets, once or twice daily after meals <https://www.chemistcounterdirect.co.uk/normacol-sachets-7g-60.html> |
| NORMACOL PLUS SCHT 60 | Sterculia | Non-stimulant | 60 |  | 2 | 30 | 1-2 sachets, once or twice daily after meals <https://www.chemistcounterdirect.co.uk/normacol-sachets-7g-60.html> |
| MICRALAX 5ML ENEMA X 12 | Sodium citrate | Non-stimulant | 12 |  | 1 | 12 | The usual dosage of this product is one tube at a time <https://www.prescriptiondoctor.com/constipation/micralax> |
| ROI Microlax rectal solution 5ml 4s (P) | Sodium citrate | Non-stimulant | 4 |  | 1 | 4 | The usual dosage of this product is one tube at a time <https://www.prescriptiondoctor.com/constipation/micralax> |
| DULCOLAX PERLES 50'S (P) | Docusate sodium | Non-stimulant | 50 |  | 4 | 12.5 | Take two to four capsules (5 mg to 10 mg) at night <https://www.expresschemist.co.uk/Dulcolax-Perles-50.html> |
| DULCOLAX PERLES 20S (G) | Docusate sodium | Non-stimulant | 20 |  | 4 | 5 | Take two to four capsules (5 mg to 10 mg) at night <https://www.expresschemist.co.uk/Dulcolax-Perles-50.html> |
| DulcoEase Soft Gel Capsules 30's (G) | Docusate sodium | Non-stimulant | 30 |  | 3 | 10 | One capsule should be taken 3 times a day to begin with <https://www.nhs.uk/medicines/docusate/how-and-when-to-take-or-use-docusate/> |
| Dulcobalance 10's (P) | Docusate sodium | Non-stimulant | 10 |  | 2 | 5 | 1 to 2 sachets (10–20g) per day <https://www.pharmacyrequirements.co.uk/dulcobalance-sachets-10sachets> |
| Pharmaceuticals Fibre Relief30 G | Ispaghula husk | Non-stimulant | 30 |  | 2 | 15 | Take 1 - 2 before you go to bed at night <https://www.senokot.co.uk/our-products/fybogel-hi-fibre-sachets/> |
| Movicol 8s (P) | Macrogol '3350' | Non-stimulant | 8 |  | 3 | 2.67 | Take 1-3 sachets a day <https://www.nhs.uk/medicines/macrogol/how-and-when-to-take-macrogol/> |
| Movicol sachets pack 30s (P) | Macrogol '3350' | Non-stimulant | 30 |  | 3 | 10 | Take 1-3 sachets a day <https://www.nhs.uk/medicines/macrogol/how-and-when-to-take-macrogol/> |
| K MOVICOL SACHET 30 | Macrogol '3350' | Non-stimulant | 30 |  | 3 | 10 | Take 1-3 sachets a day <https://www.nhs.uk/medicines/macrogol/how-and-when-to-take-macrogol/> |
| MOVICOL SACHET 20 P | Macrogol '3350' | Non-stimulant | 20 |  | 3 | 6.67 | Take 1-3 sachets a day <https://www.nhs.uk/medicines/macrogol/how-and-when-to-take-macrogol/> |
| MOVICOL HALF 7G SACH 30 | Macrogol '3350' | Non-stimulant | 30 |  | 6 | 5 | Take 2 sachets between 1 and 3 times a day depending on the severity of your constipation <https://www.chemist-4-u.com/movicol-half-powder-for-constipation-30-sachets> |
| ROI BABYLAX 3'S LAX (P) | Glycerol | Non-stimulant | 50 |  | 6 | 8.33 | Take 2 sachets between 1 and 3 times a day depending on the severity of your constipation <https://www.pharmasana.co.uk/babylax-klistier-3-st-00098878> |
| ZZ SYRP FIGS 100ML G | Figs | Non-stimulant |  | 100 | 30 | 3.33 | Take 15-30ml (one to two tablespoonfuls) <https://www.amazon.co.uk/Califig-100-ml-Syrup-Figs/dp/B01BC4A9U8?th=1> |
| #CALIFIG 55ML (G) | Figs | Non-stimulant |  | 55 | 30 | 1.83 | Take 15-30ml (one to two tablespoonfuls) <https://www.amazon.co.uk/Califig-100-ml-Syrup-Figs/dp/B01BC4A9U8?th=1> |
| Califig Syrup Of Figs food supplment 100 | Figs | Non-stimulant |  | 100 | 30 | 3.33 | Take 15-30ml (one to two tablespoonfuls) <https://www.amazon.co.uk/Califig-100-ml-Syrup-Figs/dp/B01BC4A9U8?th=1> |
| #CALIFIG 110ML (G) | Figs | Non-stimulant |  | 110 | 30 | 3.67 | Take 15-30ml (one to two tablespoonfuls) <https://www.amazon.co.uk/Califig-100-ml-Syrup-Figs/dp/B01BC4A9U8?th=1> |
| ROI Ortis Guarana Tabs 18s | Guarana | Non-stimulant | 18 |  | 1 | 18 | Take one tablet a day with plenty of liquid* |
| PICOLAX 2X10G | Sodium pico-sulfate | Non-stimulant | 2 |  | 1 | 2 | Take 1 sachet at bedtime <https://www.medicines.org.uk/emc/files/pil.915.pdf> |
| Fleet ready to use enema 133ml (P) | Sodium Phosphate | Non-stimulant | 3 |  | 1 | 3 | n/a |
| Constipation Rlf Suppositories (G) | Bisacodyl | Stimulant | 24 |  | 1 | 24 | One as required* |
| Constipation Relief Tabs 40s G | Bisacodyl | Stimulant | 40 |  | 2 | 20 | 1 to 2 tablets at night* |
| Constipation Relief Suppos 24s (G) | Bisacodyl | Stimulant | 24 |  | 1 | 24 | One as required* |
| Constipation Relief Tabs 100 (P) | Bisacodyl | Stimulant | 100 |  | 2 | 50 | 1 to 2 tablets at night* |
| NYLAX TABLETS 30'S G | Bisacodyl | Stimulant | 30 |  | 2 | 15 | 1 or 2 tablets (7.5mg or 15mg) at bedtime (source n/a) |
| MANEVAC GRANULES 400G | Isaghula Husk, Senna | Stimulant | 400 |  | 10 | 40 | 5-10g of this product should be taken once a day <https://www.dailychemist.com/product/manevac-granules-400g/> |
| Senokot Dual Relief Tablets 20's G | Sennoside | Stimulant | 20 |  | 8 | 2.5 | 4 tablets twice a day <https://senokot.com/laxatives/senokot-dual-action/> |
| Senokot Dual Relief Tablets 40's G | Sennoside | Stimulant | 40 |  | 8 | 5 | 4 tablets twice a day <https://senokot.com/laxatives/senokot-dual-action/> |
| Senokot comfort tablets 20s G | Sennoside | Stimulant | 20 |  | 8 | 2.5 | 4 tablets twice a day <https://senokot.com/laxatives/senokot-regular-strength/> |
| SENOKOT TABLETS 20S G | Sennoside | Stimulant | 20 |  | 8 | 2.5 | 4 tablets twice a day <https://senokot.com/laxatives/senokot-regular-strength/> |
| ZZ SENOKOT TABS 60S G | Sennoside | Stimulant | 60 |  | 8 | 7.5 | 4 tablets twice a day <https://senokot.com/laxatives/senokot-regular-strength/> |
| ZZ SENOKOT TABS 100 G G | Sennoside | Stimulant | 100 |  | 8 | 12.5 | 4 tablets twice a day <https://senokot.com/laxatives/senokot-regular-strength/> |
| ZZ SENOKOT MAX STR 24 (G) | Sennoside | Stimulant | 24 |  | 4 | 6 | 2 tablets twice a day <https://senokot.com/laxatives/senokot-extra-strength/> |
| SENOKOT MAX TABLETS 48 (GSL) | Sennoside | Stimulant | 48 |  | 4 | 12 | 2 tablets twice a day <https://senokot.com/laxatives/senokot-extra-strength/> |
| Senokot syrup 150ml G | Sennoside | Stimulant |  | 150 | 10 | 15 | Take one or two 5ml spoonfuls at night <https://www.senokot.co.uk/our-products/senokot-7-5-mg-5ml-syrup-12-years-plus/> |
| SENOKOT SYRUP 150ML (G) | Sennoside | Stimulant |  | 150 | 10 | 15 | Take one or two 5ml spoonfuls at night <https://www.senokot.co.uk/our-products/senokot-7-5-mg-5ml-syrup-12-years-plus/> |
| GRAVITY FED UNIT SENOKOT DUAL RLF 14S G | Sennoside | Stimulant | 14 |  | 8 | 1.75 | 4 tablets twice a day <https://senokot.com/laxatives/senokot-dual-action/> |
| SENNA TABLETS 20S G | Sennoside | Stimulant | 20 |  | 2 | 10 | Take two 7.5mg Senna Tablets at bedtime <https://www.nhs.uk/medicines/senna/how-and-when-to-take-senna/> |
| SENNA TABLETS 60S (G) | Sennoside | Stimulant | 60 |  | 2 | 30 | Take two 7.5mg Senna Tablets at bedtime <https://www.nhs.uk/medicines/senna/how-and-when-to-take-senna/> |
| SENNA TABLETS 100S (G) | Sennoside | Stimulant | 100 |  | 2 | 50 | Take two 7.5mg Senna Tablets at bedtime <https://www.nhs.uk/medicines/senna/how-and-when-to-take-senna/> |
| SENNA LAX TABS 60 G | Sennoside | Stimulant | 60 |  | 2 | 30 | Take two 7.5mg Senna Tablets at bedtime <https://www.nhs.uk/medicines/senna/how-and-when-to-take-senna/> |
| ZZ EXLAX SENNA PILL 20 (G) | Sennoside | Stimulant | 20 |  | 2 | 10 | Take two 7.5mg Senna Tablets at bedtime <https://www.nhs.uk/medicines/senna/how-and-when-to-take-senna/> |
| EX-LAX SENNA 24S | Sennoside | Stimulant | 24 |  | 2 | 12 | Take two 7.5mg Senna Tablets at bedtime <https://www.nhs.uk/medicines/senna/how-and-when-to-take-senna/> |
| TAB SENNA LAX 50 G | Sennoside | Stimulant | 50 |  | 2 | 25 | Take two 7.5mg Senna Tablets at bedtime <https://www.nhs.uk/medicines/senna/how-and-when-to-take-senna/> |
| TAB SENNA LAX 100 G | Sennoside | Stimulant | 100 |  | 2 | 50 | Take two 7.5mg Senna Tablets at bedtime <https://www.nhs.uk/medicines/senna/how-and-when-to-take-senna/> |
| ZZ NYLAX SENNA TABS 30 G | Sennoside | Stimulant | 30 |  | 2 | 15 | Take two 7.5mg Senna Tablets at bedtime <https://www.nhs.uk/medicines/senna/how-and-when-to-take-senna/> |
| EX-LAX SENNA 18 CHOC G | Sennoside | Stimulant | 18 |  | 2 | 9 | Take two 7.5mg Senna Tablets at bedtime <https://www.nhs.uk/medicines/senna/how-and-when-to-take-senna/> |
| EX-LAX SENNA 36 TABS G | Sennoside | Stimulant | 36 |  | 2 | 18 | Take two 7.5mg Senna Tablets at bedtime <https://www.nhs.uk/medicines/senna/how-and-when-to-take-senna/> |
| SENNA LAX TABS 100 G | Sennoside | Stimulant | 100 |  | 2 | 50 | Take two 7.5mg Senna Tablets at bedtime <https://www.nhs.uk/medicines/senna/how-and-when-to-take-senna/> |
| ZZ EX-LAX SENNA 48S (G) | Sennoside | Stimulant | 48 |  | 2 | 24 | Take two 7.5mg Senna Tablets at bedtime <https://www.nhs.uk/medicines/senna/how-and-when-to-take-senna/> |
| Senna tabs 20 (G) | Sennoside | Stimulant | 20 |  | 2 | 10 | Take two 7.5mg Senna Tablets at bedtime [<https://www.nhs.uk/medicines/senna/how-and-when-to-take-senna/> vqBm9wckCJO2ljBkjppCxd0](https://www.boots.com/boots-senna-7-5mg-tablets-adult-senna-pods-20-tablets-10334862?srsltid=AfmBOorSdT_6N_2TVHtrelgxiL-O4bgA-vqBm9wckCJO2ljBkjppCxd0) |
| Senna tabs 60 (G) | Sennoside | Stimulant | 60 |  | 2 | 30 | Take two 7.5mg Senna Tablets at bedtime <https://www.nhs.uk/medicines/senna/how-and-when-to-take-senna/> |
| Senna laxative tablets 20s G | Sennoside | Stimulant | 20 |  | 2 | 10 | Take two 7.5mg Senna Tablets at bedtime <https://www.nhs.uk/medicines/senna/how-and-when-to-take-senna/> |
| Senna laxative tablets 60s G | Sennoside | Stimulant | 60 |  | 2 | 30 | Take two 7.5mg Senna Tablets at bedtime <https://www.nhs.uk/medicines/senna/how-and-when-to-take-senna/> |
| Senna laxative tablets 100s G | Sennoside | Stimulant | 100 |  | 2 | 50 | Take two 7.5mg Senna Tablets at bedtime <https://www.nhs.uk/medicines/senna/how-and-when-to-take-senna/> |
| Senna tabs 100 (G) | Sennoside | Stimulant | 100 |  | 2 | 50 | Take two 7.5mg Senna Tablets at bedtime <https://www.nhs.uk/medicines/senna/how-and-when-to-take-senna/> |
| Pharma senna max laxative 24 G | Sennoside | Stimulant | 24 |  | 1 | 24 | Once a day, at bedtime <https://www.senokot.co.uk/our-products/senokot-max-strength-tablets-adult/> |
| Pharma senna max laxative 48 G | Sennoside | Stimulant | 48 |  | 1 | 48 | Once a day, at bedtime <https://www.senokot.co.uk/our-products/senokot-max-strength-tablets-adult/> |
| ZZ DULCOLAX TABS 60S (P) | Bisacodyl | Stimulant | 60 |  | 2 | 30 | Take 1 - 2 Dulcolax tablets before you go to bed at night <https://www.dulcolax.com/en-gb/products/dulcolax-adult-tablets> |
| Dulcolax tablets 60s 5mg G | Bisacodyl | Stimulant | 60 |  | 2 | 30 | Take 1 - 2 Dulcolax tablets before you go to bed at night <https://www.dulcolax.com/en-gb/products/dulcolax-adult-tablets> |
| Dulcolax Liquid 30ml G | Sodium picosulfate | Stimulant |  | 30 | 10 | 3 | Take 1-2 spoonfuls of Dulcolax Liquid at night <https://www.dulcolax.com/en-gb/products/dulcolax-pico-liquid> |
| DULCO-LAX 5MG TAB100 (P) | Bisacodyl | Stimulant | 100 |  | 2 | 50 | Take 1 - 2 Dulcolax tablets before you go to bed at night <https://www.dulcolax.com/en-gb/products/dulcolax-adult-tablets> |
| Dulcolax 40's (G) | Bisacodyl | Stimulant | 40 |  | 2 | 20 | Take 1 - 2 Dulcolax tablets before you go to bed at night <https://www.dulcolax.com/en-gb/products/dulcolax-adult-tablets> |
| ROI Dulcolax 40s | Bisacodyl | Stimulant | 40 |  | 2 | 20 | Take 1 - 2 Dulcolax tablets before you go to bed at night <https://www.dulcolax.com/en-gb/products/dulcolax-adult-tablets> |
| ZZ DULCOLAX TABS 20SG | Bisacodyl | Stimulant | 20 |  | 2 | 10 | Take 1 - 2 Dulcolax tablets before you go to bed at night <https://www.dulcolax.com/en-gb/products/dulcolax-adult-tablets> |
| DULCOLAX LIQ 100ML | Sodium picosulfate | Stimulant |  | 100 | 10 | 10 | Take 1-2 spoonfuls of Dulcolax Liquid at night <https://www.dulcolax.com/en-gb/products/dulcolax-pico-liquid> |
| DULCOLAX PICO 300ML | Sodium picosulfate | Stimulant |  | 300 | 10 | 30 | Take 1-2 spoonfuls of Dulcolax Pico Liquid at night <https://www.dulcolax.com/en-gb/products/dulcolax-pico-liquid> |
| DULCOLAX SUPPOS 12S (P) | Bisacodyl | Stimulant | 12 |  | 2 | 6 | Take 1 - 2 Dulcolax tablets before you go to bed at night <https://www.dulcolax.com/en-gb/products/dulcolax-adult-tablets> |
| Dulcolax pico liquid 100ml (P) | Sodium picosulfate | Stimulant |  | 100 | 10 | 10 | Take 1-2 spoonfuls of Dulcolax Pico Liquid at night <https://www.dulcolax.com/en-gb/products/dulcolax-pico-liquid> |
| EX-LAX CHOC LAX 18 G | Sennoside | Stimulant | 18 |  | 1 | 18 | One per day <https://www.pharmacyrequirements.co.uk/ex-lax-senna-chocolate-laxative> |
| METHYTD SPIRIT 500ML (P) | n/a | Other | - | - | - | - | n/a |
| Buscopan IBS relief tablets 40s G | n/a | Other | - | - | - | - | n/a |
| BUSCOPAN IBS RLF TAB20 (G) | n/a | Other | - | - | - | - | n/a |
| Buscopan Cramps 20's (P) | n/a | Other | - | - | - | - | n/a |
| DP Buscopan Cramps 20's (P) | n/a | Other | - | - | - | - | n/a |
| Dulcoglide applicators for use with supp | n/a | Other | - | - | - | - | n/a |
| EPSOM SA 500G E G | n/a | Other | - | - | - | - | n/a |
| Epsom Salts 200g (G) | n/a | Other | - | - | - | - | n/a |

**Source reserved to protect retailer anonymity.*
