# Supplementary material for: Using supermarket loyalty card data to investigate seasonal variation in laxative purchases in the UK
Source: PLOS Digit Health. 2026 Mar 18;5(3):e0000963. doi: 10.1371/journal.pdig.0000963 (PMC12998798; doi:10.1371/journal.pdig.0000963)
Supplement: S2 Text — (DOCX) [file pdig.0000963.s002.docx]

# S2 Comparisons of Different Buyer Groups

Our manuscript focuses on frequent buyers, defined as the top 10% of purchasers. This choice was made to ensure results reflected the most loyal and active consumers. This section presents results for other buyer groups—including the top 5%, 20%, and all buyers—for comparison.

Table 2 reports the mean (SD) overall laxative doses purchased for each group. As expected, the included buyers consistently purchased substantially higher quantities compared with excluded buyers, demonstrating clear separation between loyal customers and the broader customer population. This supports the rationale for defining buyer subgroups based on purchasing volume.

**S2 Table A:** Mean (SD) laxative doses purchased for different buyer groups.

|  | **Included** | | **Excluded** | |
| --- | --- | --- | --- | --- |
|  | **Mean** | **SD** | **Mean** | **SD** |
| Top 5% (n = 38,146) | 373.16 | 621.60 | 19.58 | 19.88 |
| Top 10% (n = 85,578) | 233.68 | 469.93 | 16.17 | 13.13 |
| Top 20% (n = 149,692) | 138.29 | 343.00 | 12.43 | 7.85 |
| All buyers (n = 748,375) | 37.60 | 161.61 | - | - |

## Top 5% of Laxative Buyers

The top 20% of laxative buyers included 38,146 individuals, comprising 547, 344 item purchases.

**S2 Table B:** Regression results for top 5% of laxative buyers.

| **Hypothesis** | **Variable** | **Coefficient** | **CI** | **p** | **p_adj** | **IRR** | **IRR_CI** |
| --- | --- | --- | --- | --- | --- | --- | --- |
| H1a | inflation constant | -1.03 | -1.05, -1.02 | <.001 | - | - | - |
|  | intercept | 4.06 | 4.04, 4.08 | <.001 | - | - | - |
|  | month (Jan = 1) | 0.00 | -0.01, 0.01 | .443 | - | 1.00 | 0.99, 1.01 |
|  | alpha | 38.85 | 36.92, 40.79 | <.001 | - | - | - |
| H1b | inflation constant | -0.36 | -0.37, -0.35 | <.001 | - | - | - |
|  | intercept | 4.04 | 4.02, 4.05 | <.001 | - | - | - |
|  | May | -0.02*** | -0.03, -0.01 | <.001 | <.001 | 0.98 | 0.97, 0.99 |
|  | June | 0.00 | -0.01, 0.01 | .723 | .723 | 1.00 | 0.99, 1.01 |
|  | July | 0.03*** | 0.02, 0.04 | <.001 | <.001 | 1.03 | 1.02, 1.04 |
|  | August | 0.03*** | 0.02, 0.04 | <.001 | <.001 | 1.03 | 1.02, 1.04 |
|  | alpha | 37.58 | 35.74, 39.41 | <.001 | - | - | - |
| H2a | Inflation constant | -0.72 | 0.74, -0.70 | <.001 | - | - | - |
|  | Intercept | 0.67 | 0.62, 0.72 | <.001 | - | - | - |
|  | Month (Jan = 1) | 0.06** | 0.03, 0.10 | .001 | - | 1.06 | 1.03, 1.10 |
|  | Type (Stim = 1) | 3.41*** | 3.36, 3.45 | <.001 | - | 30.16 | 28.79, 31.60 |
|  | Month:Type | -0.06** | -0.09, -0.02 | .001 | - | 0.94 | 0.91, 0.98 |
|  | Alpha | 41.25 | 39.43, 43.07 | <.001 | - | - | - |
| H2b | Inflation constant | -0.19 | -0.20, -0.18 | <.001 | - | - | - |
|  | Intercept | 0.60 | 0.55, 0.65 | <.001 | - | - | - |
|  | May | 0.14*** | 0.10, 0.17 | <.001 | <.001 | 1.15 | 1.11, 1.19 |
|  | June | 0.00 | -0.04, 0.04 | .974 | .999 | 1.00 | 0.96, 1.04 |
|  | July | 0.03 | -0.01, 0.06 | .157 | .783 | 1.03 | 0.99, 1.06 |
|  | August | 0.05 | 0.02, 0.09 | .003 | .016 | 1.06 | 1.02, 1.09 |
|  | Type (Stim = 1) | 3.45*** | 3.41, 3.50 | <.001 | <.001 | 31.56 | 30.12, 33.08 |
|  | May:Type | -0.16*** | -0.20, -0.12 | <.001 | <.001 | 0.85 | 0.82, 0.88 |
|  | June:Type | 0.00 | -0.04, 0.04 | .913 | .999 | 1.00 | 0.96, 1.04 |
|  | July:Type | 0.00 | -0.04, 0.04 | .947 | .999 | 1.00 | 0.96, 1.04 |
|  | August:Type | -0.03 | -0.06, 0.01 | .157 | .783 | 0.97 | 0.94, 1.01 |
|  | Alpha | 39.88 | 38.15, 41.60 | <.001 | - | - | - |

*<.05, **<.01, ***<.001. p_adj refers to Holm-adjusted p values; IRR refers to Incidence Rate Ratios; IRR_CI refers to the 95% confidence interval for the IRR.

For H1a, there was no evidence of a difference in stimulant laxative purchases between December and January. For H1b, seasonal variation was observed: around 2% fewer doses were purchased in May compared to September, and around 3% more doses were purchased in July and August.

For H2a, purchases of stimulant doses were largely higher than non-stimulants overall. January purchases were 6% higher than December, but the increase was less pronounced for stimulants. For H2b, seasonal effects varied by laxative type. Around 15% more laxative doses were purchased in May and 6% in August overall, but stimulant purchases increased less compared to non-stimulants in May (and showed no clear change in June, July, or August).

## Results for Top 20% of Laxative Buyers

The top 20% of laxative buyers included 149,692 individuals, comprising 939,489 item purchases.

**S2 Table C:** Regression results for top 20% of laxative buyers.

| **Hypothesis** | **Variable** | **Coefficient** | **CI** | **p** | **p_adj** | **IRR** | **IRR_CI** |
| --- | --- | --- | --- | --- | --- | --- | --- |
| H1a | inflation constant | -0.60 | -0.60, -0.59 | <.001 | - | - | - |
|  | intercept | 3.70 | 3.69, 3.72 | <.001 | - | - | - |
|  | month (Jan = 1) | 0.00 | -0.01, 0.01 | .570 | - | 1.00 | 0.99, 1.00 |
|  | alpha | 30.01 | 28.84, 31.17 | <.001 | - | - | - |
| H1b | inflation constant | 0.41 | 0.40, 0.42 | <.001 | - | - | - |
|  | intercept | 3.69 | 3.67, 3.70 | <.001 | - | - | - |
|  | May | -0.03*** | -0.03, -0.02 | <.001 | <.001 | 0.97 | 0.97, 0.98 |
|  | June | 0.00 | -0.01, 0.01 | .971 | .971 | 1.00 | 0.99, 1.01 |
|  | July | 0.02*** | 0.01, 0.03 | <.001 | <.001 | 1.02 | 1.01, 1.03 |
|  | August | 0.03*** | 0.02, 0.03 | <.001 | <.001 | 1.03 | 1.02, 1.03 |
|  | alpha | 29.43 | 28.32, 30.53 | <.001 | - | - | - |
| H2a | Inflation constant | -0.10 | -0.11, -0.09 | <.001 | - | - | - |
|  | Intercept | 1.18 | 1.14, 1.21 | <.001 | - | - | - |
|  | Month (Jan = 1) | 0.11*** | 0.08, 0.13 | <.001 | - | 1.11 | 1.08, 1.14 |
|  | Type (Stim = 1) | 2.54*** | 2.51, 2.57 | <.001 | - | 12.72 | 12.35, 13.10 |
|  | Month:Type | -0.11*** | -0.14, -0.08 | <.001 | - | 0.90 | 0.87, 0.92 |
|  | Alpha | 31.94 | 30.94, 32.95 | <.001 | - | - | - |
| H2b | Inflation constant | 0.71 | 0.70, 0.72 | <.001 | - | - | - |
|  | Intercept | 1.20 | 1.16, 1.23 | <.001 | - | - | - |
|  | May | 0.17*** | 0.14, 0.20 | <.001 | <.001 | 1.19 | 1.15, 1.22 |
|  | June | -0.03 | -0.06, 0.00 | .040 | .159 | 0.97 | 0.94, 1.00 |
|  | July | 0.02 | -0.01, 0.04 | .279 | .558 | 1.02 | 0.99, 1.04 |
|  | August | 0.07*** | 0.04, 0.10 | <.001 | <.001 | 1.07 | 1.04, 1.10 |
|  | Type (Stim = 1) | 2.50*** | 2.47, 2.53 | <.001 | <.001 | 12.23 | 11.87, 12.60 |
|  | May:Type | -0.20*** | -0.23, -0.17 | <.001 | <.001 | 0.82 | 0.80, 0.85 |
|  | June:Type | 0.03 | 0.00, 0.06 | .048 | .159 | 1.03 | 1.00, 1.06 |
|  | July:Type | 0.00 | -0.03, 0.03 | .786 | .786 | 1.00 | 0.98, 1.03 |
|  | August:Type | -0.04* | -0.07, -0.01 | .003 | .017 | 0.96 | 0.93, 0.99 |
|  | Alpha | 31.07 | 30.12, 32.03 | <.001 | - | - | - |

*<.05, **<.01, ***<.001. p_adj refers to Holm-adjusted p values; IRR refers to Incidence Rate Ratios; IRR_CI refers to the 95% confidence interval for the IRR.

For H1a, there was no evidence of a difference in stimulant laxative purchases between December and January. For H1b, seasonal variation was observed: around 3% fewer doses were purchased in May compared to September, with no difference in June, and 2-3% more doses were purchased in July and August.

For H2a, purchases of stimulant doses were higher than non-stimulants overall. January purchases were around 11% higher than December, but this increase was less pronounced for stimulants. For H2b, seasonal effects varied by laxative type. Overall, around 19% more doses were purchased in May, and 7% more in August, but stimulant purchases increased less compared to non-stimulants in May and August. No seasonal effects were observed in June or July.

## Results for All Laxative Buyers

The total number of laxative buyers was 748,375 individuals, comprising 1,683,712 items.

**S2 Table D:** Regression results for all laxative buyers.

| **Hypothesis** | **Variable** | **Coefficient** | **CI** | **p** | **p_adj** | **IRR** | **IRR_CI** |
| --- | --- | --- | --- | --- | --- | --- | --- |
| H1a | inflation constant | -0.38 | -0.39, -0.37 | <.001 | - | - | - |
|  | intercept | 3.21 | 3.20, 3.22 | <.001 | - | - | - |
|  | month (Jan = 1) | 0.02*** | 0.01, 0.03 | <.001 | - | 1.02 | 1.01, 1.03 |
|  | alpha | 27.19 | 26.36, 28.02 | <.001 | - | - | - |
| H1b | inflation constant | 0.89 | 0.89, 0.90 | <.001 | - | - | - |
|  | intercept | 3.19 | 3.18, 3.20 | <.001 | - | - | - |
|  | May | -0.03*** | -0.04, -0.02 | <.001 | <.001 | 0.97 | 0.96, 0.98 |
|  | June | -0.01** | -0.02, 0.00 | .003 | .003 | 0.99 | 0.98, 1.00 |
|  | July | 0.04*** | 0.03, 0.05 | <.001 | <.001 | 1.04 | 1.04, 1.05 |
|  | August | 0.03*** | 0.03, 0.04 | <.001 | <.001 | 1.03 | 1.03, 1.04 |
|  | alpha | 27.48 | 26.69, 28.27 | <.001 | - | - | - |
| H2a | Inflation constant | 0.40 | 0.39, 0.41 | <.001 | - | - | - |
|  | Intercept | 1.67 | 1.65, 1.70 | <.001 | - | - | - |
|  | Month (Jan = 1) | 0.02 | 0.00, 0.04 | .060 | - | 1.02 | 1.00, 1.04 |
|  | Type (Stim = 1) | 1.53*** | 1.51, 1.55 | <.001 | - | 4.63 | 4.53, 4.73 |
|  | Month:Type | 0.00 | -0.03, 0.02 | .810 | - | 1.00 | 0.97, 1.02 |
|  | Alpha | 25.01 | 24.40, 25.63 | <.001 | - | - | - |
| H2b | Inflation constant | 1.40 | 1.39, 1.40 | <.001 | - | - | - |
|  | Intercept | 1.56 | 1.54, 1.58 | <.001 | - | - | - |
|  | May | 0.14*** | 0.12, 0.16 | <.001 | <.001 | 1.15 | 1.12, 1.18 |
|  | June | -0.09*** | -0.11, -0.06 | <.001 | <.001 | 0.92 | 0.90, 0.94 |
|  | July | 0.00 | -0.02, 0.03 | .680 | .680 | 1.00 | 0.98, 1.03 |
|  | August | 0.12*** | 0.10, 0.15 | <.001 | <.001 | 1.13 | 1.11, 1.16 |
|  | Type (Stim = 1) | 1.62*** | 1.60, 1.64 | <.001 | <.001 | 5.07 | 4.96, 5.18 |
|  | May:Type | -0.17*** | -0.19, -0.15 | <.001 | <.001 | 0.84 | 0.82, 0.86 |
|  | June:Type | 0.08*** | 0.05, 0.10 | <.001 | <.001 | 1.08 | 1.06, 1.11 |
|  | July:Type | 0.03* | 0.01, 0.06 | .008 | .015 | 1.03 | 1.01, 1.06 |
|  | August:Type | -0.10*** | -0.12, -0.07 | <.001 | <.001 | 0.91 | 0.88, 0.93 |
|  | Alpha | 25.63 | 25.03, 26.23 | <.001 | - | - | - |

*<.05, **<.01, ***<.001. p_adj refers to Holm-adjusted p values; IRR refers to Incidence Rate Ratios; IRR_CI refers to the 95% confidence interval for the IRR.

For H1a, there was evidence that stimulant laxative purchases were 2% higher in January compared with December. For H1b, relative to September, purchases were 3% lower in May, 4% higher in July, and 3% higher in August, with no meaningful change in June.

For H2a, stimulant laxative purchases were substantially higher than non-stimulants, at 4.6 times the expected number of doses, with no additional effect of January over December. For H2b, compared with September, purchases were 15% higher in May, 13% higher in August, and 8% lower in June. Interaction terms with laxative type indicated that seasonal increases were generally smaller for stimulants, reflecting that non-stimulant purchases contributed proportionally more to the summer peaks.

## Overview of Similarities and Differences

Across buyer groups, seasonal patterns in laxative purchasing were generally consistent, though the magnitude of effects vary.

For H1a, there was no meaningful change for the top 5%, 10%, or 20% buyers, whereas for all buyers, purchases were 2% higher in January. For H1b, dose purchases were 2–4% higher in July and August and 2–3% lower in May across all buyer groups, while June generally showed no seasonal effect (except for a 1% decrease for all buyers). For H2a, stimulant dose purchases were substantially higher than non-stimulants across all groups: around 12–30 times higher for the top 5%, 10% and 20% of buyers, and 4.6 times higher for all buyers. Interaction terms indicated that the January effect was slightly stronger for non-stimulants among most groups (except all buyers). For H2b, non-stimulant doses exhibited larger proportional increases during summer months across all top buyer groups, particularly in May (15–20% higher) and August (5–13% higher), whereas stimulant doses were either similar to September or slightly lower. When examining all buyers, most of these summer effects were still statistically significant, although the magnitude was smaller due to the larger proportion of low-volume purchasers. These patterns indicate that the seasonal trends are broadly consistent across groups, but effect sizes are amplified among higher-volume buyers.

Together, these comparisons suggest that the top 10% of buyers provide a clear and interpretable signal of seasonal variation and type-specific effects, balancing meaningful effect sizes with sufficient sample stability. In contrast, using all buyers appears to inflate statistical significance due to the large number of low-volume purchasers, while focusing only on the top 5% reduces generalisability. Accordingly, the top 10% was selected for the main analyses.
