# Supplementary material for: Using supermarket loyalty card data to investigate seasonal variation in laxative purchases in the UK
Source: PLOS Digit Health. 2026 Mar 18;5(3):e0000963. doi: 10.1371/journal.pdig.0000963 (PMC12998798; doi:10.1371/journal.pdig.0000963)
Supplement: S3 Text — (DOCX) [file pdig.0000963.s003.docx]

**S3 Results using Sales Units**

As a point of comparison, we present our regression analyses using sales units as the outcome (as opposed to dosage), again limited to the top 10% of laxative buyers (based on sales units). This gives 85,578 buyers, covering 817,061 items purchased.

| **Hypothesis** | **Variable** | **Coefficient** | **CI** | **p** | **p_adj** | **IRR** | **IRR_CI** |
| --- | --- | --- | --- | --- | --- | --- | --- |
| H1a | inflation constant | -0.85 | -0.86, -0.84 | <.001 | - | - | - |
|  | intercept | 3.74 | 3.72, 3.75 | <.001 | - | - | - |
|  | month (Jan = 1) | 0.00 | -0.01, 0.01 | .733 | - | 1.00 | 0.99, 1.01 |
|  | alpha | 41.31 | 39.61, 43.00 | <.001 | - | - | - |
| H1b | inflation constant | -0.01 | -0.02, 0.00 | .014 | - | - | - |
|  | intercept | 3.70 | 3.69, 3.72 | <.001 | - | - | - |
|  | May | -0.01* | -0.02, -0.01 | .002 | .003 | 0.99 | 0.98, 0.99 |
|  | June | 0.00 | -0.01, 0.01 | .748 | .748 | 1.00 | 0.99, 1.01 |
|  | July | 0.04*** | 0.03, 0.04 | <.001 | <.001 | 1.04 | 1.03, 1.04 |
|  | August | 0.03*** | 0.02, 0.04 | <.001 | <.001 | 1.03 | 1.02, 1.04 |
|  | alpha | 40.21 | 38.62, 41.80 | <.001 | - | - | - |
| H2a | Inflation constant | -0.18 | -0.20, -0.17 | <.001 | - | - | - |
|  | Intercept | 1.52 | 1.48, 1.55 | <.001 | - | - | - |
|  | Month (Jan = 1) | 0.11*** | 0.08, 0.14 | <.001 | - | 1.12 | 1.09, 1.15 |
|  | Type (Stim = 1) | 2.22*** | 2.18, 2.25 | <.001 | - | 9.17 | 8.89, 9.47 |
|  | Month:Type | -0.11*** | -0.14, -0.08 | <.001 | - | 0.89 | 0.87, 0.92 |
|  | Alpha | 40.76 | 39.37, 42.14 | <.001 | - | - | - |
| H2b | Inflation constant | 0.43 | 0.42, 0.44 | <.001 | - | - | - |
|  | Intercept | 1.50 | 1.47, 1.54 | <.001 | - | - | - |
|  | May | 0.18*** | 0.15. 0.21 | <.001 | <.001 | 1.20 | 1.16, 1.23 |
|  | June | 0.01 | -0.02, 0.04 | .692 | .999 | 1.01 | 0.98, 1.04 |
|  | July | 0.08*** | 0.05, 0.10 | <.001 | <.001 | 1.08 | 1.05, 1.11 |
|  | August | 0.08*** | 0.05, 0.10 | <.001 | <.001 | 1.08 | 1.05, 1.11 |
|  | Type (Stim = 1) | 2.20*** | 2.17, 2.23 | <.001 | <.001 | 9.00 | 8.72, 9.30 |
|  | May:Type | -0.20*** | -0.23, -0.17 | <.001 | <.001 | 0.82 | 0.80, 0.85 |
|  | June:Type | -0.01 | -0.04, 0.02 | .610 | .999 | 0.99 | 0.96, 1.02 |
|  | July:Type | -0.04 | -0.07, -0.01 | .006 | .018 | 0.96 | 0.93, 0.99 |
|  | August:Type | -0.04 | -0.08, -0.01 | .005 | .018 | 0.96 | 0.93, 0.99 |
|  | Alpha | 39.49 | 38.19, 40.79 | <.001 | - | - | - |

**S3 Table A:** Regression results using sales units as the outcome.

*<.05, **<.01, ***<.001. p_adj refers to Holm-adjusted p values; IRR refers to Incidence Rate Ratios; IRR_CI refers to the 95% confidence interval for the IRR.

Analyses using sales units largely mirrored the results obtained with doses. For H1a, there was no meaningful difference in purchases between January and December, consistent with the dose-based analysis. For H1b, purchases were slightly lower in May (1% below September) and higher in July (4%) and August (3%), closely matching the patterns observed for doses, with effect directions consistent and magnitudes differing by only 1–2%.

For H2a, stimulant sales were substantially higher than non-stimulants, around 9.2 times higher for sales units compared to 17.9 for doses, reflecting smaller proportional differences when measured in units. Interaction terms in both cases indicated that January effects were slightly stronger for non-stimulants. For H2b, non-stimulant sales exhibited proportional increases during summer months (May: 20% higher; August: 8% higher), while stimulant sales were similar or slightly reduced relative to September, closely mirroring patterns observed for doses. However, non-stimulant sales showed slightly attenuated effects in July compared to doses purchased (8% higher versus no effect).

Overall, these results show that seasonal effects are consistent across measurement units, with only minor attenuation in sales units compared to doses. As doses are likely to be a more accurate reflection of consumption, they remain the preferred metric for capturing these purchasing patterns.
