# Supplementary material for: Using supermarket loyalty card data to investigate seasonal variation in laxative purchases in the UK
Source: PLOS Digit Health. 2026 Mar 18;5(3):e0000963. doi: 10.1371/journal.pdig.0000963 (PMC12998798; doi:10.1371/journal.pdig.0000963)
Supplement: Attachment — Submitted filename: PDIG-D-25-00477-reivew.docx [file pdig.0000963.s004.docx]

**Summary**

The manuscript analyzes 2013–2014 UK loyalty-card transactions from a national pharmacy chain to test existing hypotheses about seasonal variations in laxative purchases, specifically the claimed peaks in January (“New Year’s resolutions”) and summer (“appearance”). It also compares patterns between stimulant and non-stimulant laxatives and with weight-management products. The authors use zero-inflated negative binomial regression (ZINB) with a dosage-based outcome for laxatives (maximum dosage duration) and sales-unit outcomes for comparison categories, focusing on the top 10% of laxative buyers, with additional analyses on all buyers.

**Major comments**

1. Causal language and strength of inference: The paper often shifts from seasonality and weight-control motivation. While the triangulation with weight-management product seasonality is suggestive, alternative explanations (such as GI infection seasonality, travel-related constipation, hydration/heat effects, promotions, and store traffic) remain plausible. Please temper causal language (e.g., “aligned with,” “may suggest”) throughout and expand the discussion of alternative mechanisms and how your negative controls do (and do not) rule them out. Consider falsification tests using additional OTC categories with similar price/promo dynamics but no body-image link.
2. Sampling frame: “top 10%” buyers: Limiting primary analyses to the top decile of buyers risks selection on the dependent variable and may overrepresent chronic constipation or idiosyncratic shoppers. Provide a priori justification for the 10% threshold, and a data-driven sensitivity grid (e.g., top 5/10/20/30%) showing whether seasonal IRRs are stable across cutoffs. Buyer-level descriptives (e.g., median monthly purchases, interquartile ranges) for the included vs excluded populations. Clear motivation for why “frequent buyers” better represent the mechanisms of interest, given the hypothesis focuses on seasonal (time-varying) behavior, not high levels of purchasing.
3. Modeling strategy & panel structure: You have longitudinal, person-month counts with many zeros. Should the generalized mixed effect model or GEE models need to be used to account for within-subject correlation?
4. Cluster standard errors at the buyer (and possibly store-region) level. If retaining ZINB, specify (i) the variables in the zero-inflation equation, (ii) goodness-of-fit vs NB/Poisson (Vuong, AIC/BIC), and (iii) whether zero inflation is buyer- or structure-driven. Report incidence rate ratios (IRRs) with CIs in tables/figures; they are much easier to interpret than raw coefficients.
5. Controls for promotions and holidays: OTC sales are highly responsive to promotions, leaflets, and seasonal events (e.g., Christmas, summer travel). Please indicate whether you can proxy for these and discuss promotion confounding in the limitations section.
6. Product classification and dosage assumptions: The stimulant/non-stimulant taxonomy is hand-labeled. Provide a codebook (supplement) listing each SKU, active ingredient(s), assigned class, quantity, and dosage assumptions, plus inter-rater checks if applicable. Some items listed as non-stimulant (e.g., guarana, syrup of figs) may be supplements or mixed-purpose—please justify inclusion/exclusion and test robustness, excluding ambiguous products. The maximum adult dose assumption could overstate “doses” vs typical use. Add sensitivity analyses using the recommended starting dose and median dose assumptions where available.
7. Negative controls and expected seasonality: The text states little/no seasonality expected for painkillers, but Fig. 1 (as described) suggests some winter increase; reconcile the expectation with observed patterns and discuss whether that weakens/strengthens your argument. Will adding an unrelated personal-care category (e.g., shampoo) as a “pure retail” control for store traffic seasonality be helpful?
8. Weight-management co-purchase analysis: The reported Pearson correlation (r≈0.11 among overlapping buyers) is weak and ignores time order.
9. Generalizability & time period: Data are from Dec 2013–Dec 2014 and from a single retailer with ~87% female cardholders. Discuss how this affects external validity (gender skew; chain clientele), and whether patterns persist in later years. Please temper any policy-impact language accordingly.
10. Multiple testing/hypothesis structure: You test several month contrasts (Jan vs Dec; May–Aug vs Sep; interactions by type; multiple categories). Clarify your pre-specified contrasts, control the family-wise error rate (e.g., Holm) or FDR, and present adjusted p-values where appropriate.
