# Supplementary material for: Using supermarket loyalty card data to investigate seasonal variation in laxative purchases in the UK
Source: PLOS Digit Health. 2026 Mar 18;5(3):e0000963. doi: 10.1371/journal.pdig.0000963 (PMC12998798; doi:10.1371/journal.pdig.0000963)
Supplement: Attachment — Submitted filename: Response to Reviewers.docx [file pdig.0000963.s006.docx]

**Dear Editors and Reviewers,**

Thank you for your insightful comments and the opportunity to revise our paper for publication in *PLOS Digital Health*. We have made significant changes to implement these suggestions and we believe this has resulted in an improved paper that meets the publication criteria.

Our changes are summarised in the table below, including page, table, and figure numbers reflective of the revised version of the manuscript.

Sincerely,

The Authors

| **Topic / Comment(s)** | **Response** |
| --- | --- |
| **Language, Interpretation, Causal Claims** |  |
| The paper often shifts from seasonality and weight-control motivation. While the triangulation with weight-management product seasonality is suggestive, alternative explanations (such as GI infection seasonality, travel-related constipation, hydration/heat effects, promotions, and store traffic) remain plausible. Please temper causal language (e.g., “aligned with,” “may suggest”) throughout. Consider falsification tests using additional OTC categories with similar price/promo dynamics but no body-image link. [R1]  Some statements imply stronger causal conclusions than the data support (e.g., that purchases demonstrate misuse or that the findings directly support MHRA policy). Please temper this language. It would be more accurate to state that the findings are consistent with seasonal patterns that may reflect weight-control behaviours, while acknowledging that intent cannot be confirmed. [R3] | We have tempered causal language throughout the manuscript as appropriate:  *“[…] which may be consistent with seasonal patterns in weight-related behaviours* ” [line 28]  *“[…] which may suggest motivations linked to body image*” [line 219]  We have also reaffirmed in multiple instances that intent cannot be confirmed:  “*these patterns provide only indirect evidence and cannot confirm underlying intent (like body image concerns)”* [line 31]  “*However, […] this should be interpreted cautiously, as we cannot confirm actual use or intent behind purchasing*.” [line 255] |
| The discussion linking findings to MHRA's stimulant laxative regulations is interesting, but the evidence presented does not show stimulants as the main driver of seasonality. The policy relevance could be reframed more broadly, highlighting how loyalty card data can inform public health interventions and be used to evaluate the impact of regulatory measures over time. [R2] | We have reframed the MHRA/policy discussion, ensuring that we do not overstate the strength of our evidence.  *“While the evidence is not strong enough to directly validate the MHRA laxative regulation, our findings highlight an opportunity for future studies to evaluate seasonal trends in laxative use post-regulation, thereby assessing the policy impact over time.”* [line 277]  We have kept the discussion about policy more broad, and added the following point about using loyalty cards for providing policy evidence:  “*In addition, this demonstrates how loyalty card data can support public health interventions and provide a scalable resource for evaluating policy measures over time in real-world settings.”* [line 279] |
| The abstract slightly overstates conclusions by attributing observed purchasing patterns directly to body image concerns; this should be softened to reflect the indirect nature of the evidence. [R2] | We have softened our conclusions in the abstract: *“[…] which may be consistent with seasonal patterns in weight-related behaviours* ” [line 28]  We have also highlighted the indirect nature of the evidence: “*[…] these patterns provide only indirect evidence and cannot confirm underlying intent (like body image concerns)”* [line 31] |
| While the triangulation with weight-management product seasonality is suggestive, alternative explanations (such as GI infection seasonality, travel-related constipation, hydration/heat effects, promotions, and store traffic) remain plausible. [R1]  Purchase data alone cannot confirm intent. Expanding discussion of alternative explanations (e.g., travel-related constipation, dietary changes, dehydration) would provide a more balanced interpretation. [R2] | We have expanded our discussion of alternative explanations for seasonal patterns.  “*However, there are notable alternative explanations that cannot be ruled out, as purchasing of laxative and weight management products may be influenced by unrelated health (travel-related constipation, gastrointestinal infections) or retail (in-store traffic, promotions) factors.”* [line 228]  “*Nevertheless, these controls cannot eliminate all alternative explanations, particularly those specific to gastrointestinal conditions or behaviours.”* [line 240]  This has also been addressed by adding shampoo as a control for store traffic (see below). |
| **Seasonal and Retail Confounders** |  |
| Controls for promotions and holidays: OTC sales are highly responsive to promotions, leaflets, and seasonal events (e.g., Christmas, summer travel). Please indicate whether you can proxy for these and discuss promotion confounding in the limitations section. [R1]  Seasonal retailer promotions, holiday sales, and overall transaction volume could influence purchasing patterns. Please either adjust for these factors statistically or expand the limitations section to quantify their possible impact. [R3] | The impact of seasonality and promotions on sales is an ongoing difficulty in loyalty card research, and one for which there is not yet a strong answer. In some previous studies, data surrounding the Christmas/Festive period has been removed from analyses, however that was not viable in this case as December was exactly the month in question for our hypothesis.  However, we have expanded the limitations to highlight this, including the value of our shampoo control group (see below), and quantifying the potential impact on our results:  “*Seasonal retailer activity, including promotions, holiday sales periods, and fluctuations in overall store traffic, may also influence purchasing patterns. Although we could not adjust directly for promotional calendars, our shampoo negative control suggests these factors are unlikely to account for the observed effects in laxative purchasing; the variation in shampoo sales did not mirror those of laxatives, indicating that general store traffic alone is insufficient to explain the peaks. In addition, the impact of promotions and increased purchasing during the festive period is an ongoing concern in loyalty card research. While previous studies have addressed this by excluding these weeks from analyses (21), this approach was infeasible in the present study as December was a key month of interest for our hypotheses; consequently, some results may reflect a small upward bias from holiday-related increases in purchasing. Nonetheless, developing analytic techniques to account for these confounding influences will help provide a more accurate understanding of their impact on the sales of health-focused products.”* [line 310] |
| Expand the discussion of alternative mechanisms and how your negative controls do (and do not) rule them out. [R1] | Following our addition of alternative mechanisms to our discussion (in-store traffic, GI problems, travel etc), we have added clarification for how negative controls may rule out some of these explanations.  “*Even so, our negative controls provide strong evidence that the observed fluctuations are not simply reflections of broader seasonal or retail-driven patterns. Hay fever and cold/flu medications followed expected peaks in winter and spring, respectively, confirming that our approach is sensitive to known seasonal effects. Painkillers showed a modest winter increase, contrary to our expectation of minimal seasonality; but this is consistent with known rises in winter* *illnesses and therefore does not weaken our interpretation. Shampoo, included as a non–health-related control, shows no seasonality and instead fluctuates randomly throughout the year, typical of general consumer behaviour.*  *Together, these controls suggest that products known to show predictable seasonality do so in our data, and those without expected seasonality do not. This strengthens the inference that the distinctive patterns in laxative and weight-management purchasing are not attributable to general retail seasonality or broad health-related cycles.”* [line 230] |
| The text states little/no seasonality expected for painkillers, but Fig. 1 (as described) suggests some winter increase; reconcile the expectation with observed patterns and discuss whether that weakens/strengthens your argument. [R1] | We have added a point to clarify the difference in observed painkiller trends, and how this does not take away from our negative controls as the pattern is still distinct (both from laxatives/weight management), meaning this doesn’t reflect general seasonality:  “*Painkillers showed a modest winter increase, contrary to our expectation of minimal seasonality; but this is consistent with known rises in winter* *illnesses and therefore does not weaken our interpretation. […]*  *Together, these controls suggest that products known to show predictable seasonality do so in our data, and those without expected seasonality do not. This strengthens the inference that the distinctive patterns in laxative and weight-management purchasing are not attributable to general retail seasonality or broad health-related cycles.”* [line 233] |
| Will adding an unrelated personal-care category (e.g., shampoo) as a “pure retail” control for store traffic seasonality be helpful? [R1] | We have added shampoo as a “pure retail” control, to help us unpack any effects of store traffic seasonality (see Figure 1).  This is highlighted in the introduction:  *“In addition, shampoo sales are included to capture overall seasonal trends in in-store traffic, as these should otherwise be fairly consistent throughout the year.”* [line 97]  The results:  *“Mean product sales per month were calculated between December 2013 and December 2014 for the top 10% of buyers in six product categories—laxatives (n = 85,578 buyers), […] and shampoo (n = 626,501 buyers).”* [line 109]  “*Shampoo sales fluctuate throughout the year, with peaks in March, May/June, and September.”* [line 120]  And in the discussion:  “*Shampoo, included as a non–health-related control, shows no seasonality and instead fluctuates randomly throughout the year, typical of general consumer behaviour.*  *Together, these controls suggest that products known to show predictable seasonality do so in our data, and those without expected seasonality do not. This strengthens the inference that the distinctive patterns in laxative and weight-management purchasing are not attributable to general retail seasonality or broad health-related cycles.”* [line 235] |
| **Sampling Frame and Buyer selection** |  |
| Justify the decision to focus on the “top 10%” of frequent buyers and show that findings are robust across alternative thresholds (e.g., top 5%, 20%, and all buyers). At minimum, integrate results for all buyers into the main text. [R3]  -------------------------------------------------------  “top 10%” buyers: Limiting primary analyses to the top decile of buyers risks selection on the dependent variable and may overrepresent chronic constipation or idiosyncratic shoppers. Provide a priori justification for the 10% threshold, and a data-driven sensitivity grid (e.g., top 5/10/20/30%) showing whether seasonal IRRs are stable across cutoffs. Clear motivation for why “frequent buyers” better represent the mechanisms of interest, given the hypothesis focuses on seasonal (time-varying) behavior, not high levels of purchasing. [R1]  Restricting the main analysis to the top 10% of buyers is a defensible strategy, but the rationale for selecting this specific threshold should be more clearly explained. Sensitivity analyses using alternative cut-offs (e.g., top 5%, top 25%) would strengthen the robustness of the conclusions. [R2] | We provide a justification for our choice to focus on the top 10% of buyers:  “*This threshold was chosen a priori to balance sample size with capturing individuals with the highest purchasing behaviour, who are most likely to include potential misuse cases*.” [line 418]  “*In the context of laxatives, frequent buyers may reflect habitual purchasing for ongoing health concerns (e.g., digestive issues), or, in some cases, misuse related to weight management, particularly for stimulant laxatives.”* [line 414]  In addition, we have added sensitivity analyses with alternative thresholds (top 5%, top 20%, all buyers) to show that findings are robust across groups. These are presented in the Supplementary Material but are discussed in the Methods:  “*To evaluate the robustness of this choice, we conducted sensitivity analyses using alternative thresholds (top 5% and 20% of buyers), as well as analyses including all buyers (see Supplementary Material). […] These sensitivity checks confirmed that the direction and general pattern of seasonal effects were consistent across all thresholds, however, the top 10% group captured clear seasonal peaks while avoiding dilution of effects seen in the larger sample sizes (i.e., which likely reflected inflated statistical significance due to the large sample size).”* [line 420] |
| Buyer-level descriptives (e.g., median monthly purchases, interquartile ranges) for the included vs excluded populations. [R1] | Alongside our added analyses for the top 5%, 20% and all buyers, we provide descriptive statistics for the included vs excluded populations in each case (mean doses purchased and SD). These are reported in full in the Supplementary Material.  “*We also provide descriptive statistics in each case comparing included and excluded buyers.”* [line 421]  In the Supplementary Material, this is discussed:  “*Table 2 reports the mean (SD) overall laxative doses purchased for each group. As expected, the included buyers consistently purchased substantially higher quantities compared with excluded buyers, demonstrating clear separation between loyal customers and the broader customer population. This supports the rationale for defining buyer subgroups based on purchasing volume.”* [line 10] |
| **Modelling and Statistical Analyses** |  |
| The choice of modelling framework is appropriate, but important details are missing. Please report model specifications (link function, offsets, clustering by customer or store, diagnostics, handling of multiple comparisons). [R3]  --------------------  Cluster standard errors at the buyer (and possibly store-region) level. If retaining ZINB, specify (i) the variables in the zero-inflation equation, (ii) goodness-of-fit vs NB/Poisson (Vuong, AIC/BIC), and (iii) whether zero inflation is buyer- or structure-driven. [R1]  You have longitudinal, person-month counts with many zeros. Should the generalized mixed effect model or GEE models need to be used to account for within-subject correlation? [R1] | We made the decision to retain the use of ZINB models, as per the suggestion of R2. However, as per R1, we have:  **Specified the model specifications:**  “*An offset term of log(total laxative units purchased) was included to adjust for differences in individual purchasing exposure. The model was estimated using maximum likelihood via the BFGS optimiser with up to 1,000 iterations.”* [line 444]  “[…] *fitted with a logit link function, modelling the probability of a “structural zero” (i.e., cases where no purchase occurs because the customer did not buy stimulant laxatives during that period).”* [line 439]  **Used Vuong tests and AIC/BIC values to confirm that ZINB provided a superior fit relative to Poisson and standard negative binomial alternatives;**  “*Model fit is evaluated using Vuong tests comparing the ZINB model against standard negative binomial and Poisson models. Relative model performance was also assessed using Akaike (AIC) and Bayesian (BIC) information criteria, with lower values indicating better fit.”* [line 448]  For example (Hypothesis 1):  “*Model fit comparisons showed that the zero-inflated negative binomial (ZINB) model consistently outperformed standard negative binomial (NB) and Poisson alternatives. For H1a, the ZINB model achieved the lowest AIC/BIC values (AIC = 652,627.40; BIC = 652,664.71) relative to NB (AIC = 684,920.99; BIC = 684,948.98) and Poisson (AIC = 4,841,093.91; BIC = 4,841,112.57). For H1b, the same pattern was observed (ZINB AIC = 1,783,666.11; BIC = 1,783,740.27; NB AIC = 1,895,763.84; BIC = 1,895,827.40; Poisson AIC = 16,594,493.46; BIC = 16,594,546.43). Vuong tests further supported the ZINB model (H1a: Z = 73.64 vs NB, 61.42 vs Poisson; H1b: Z = 134.4 vs NB, 118.4 vs Poisson; all p < 0.001), confirming that inclusion of a zero-inflation term provided the best fit to the data*.” [line 143]  See line 167 and 194 for other AIC/BIC descriptions.  **Adapted our analyses to ensure SEs are clustered at the buyer level;**  We have updated our analyses so that regression models now use standard errors clustered at the buyer level:  “*To account for repeated observations within individuals, standard errors are clustered at the buyer level*.” [line 446]  Results have been adjusted accordingly. |
| Present effect sizes in natural units (e.g., % change or mean additional doses purchased) with confidence intervals, in addition to regression coefficients. [R3]  Report incidence rate ratios (IRRs) with CIs in tables/figures; they are much easier to interpret than raw coefficients. [R1] | As per R1, we have revised the manuscript to report incidence rate ratios (IRRs) along with 95% confidence intervals, on top of the raw regression coefficients.  This is described in the methods:  “*For ease of interpretation, all regression tables report incidence rate ratios (IRRs) alongside the raw model coefficients. Because the count component of the ZINB model uses a log link function, each coefficient represents a proportional change in the expected number of doses purchased. We therefore exponentiated the coefficients to obtain IRRs, which express the change in expected purchases associated with each predictor:*   - *IRR > 1 indicates an increase in expected dose purchases (e.g., IRR = 1.03 represents a 3% increase in doses purchased);* - *IRR < 1 indicates a decrease in expected dose purchases (e.g., IRR = 0.97 represents a 3% decrease in doses purchased).”* [line 453]   The IRRs and IRR Cis are reflected in Tables 3-5, and interpreted plainly throughout the results. For example:  “*However, stimulant dose purchases were around 2% higher in July, 3% higher in August, and 3% lower in May compared to September (H1b).”* [line 140]  “*For H2b, purchases were 20% higher in May and 8% higher in August compared to September*.” [line 159] |
| The use of zero-inflated negative binomial regression is appropriate for these data. However, the interpretation of the inflation component is not clearly explained. Providing a brief, plain-language explanation of how the inflation term contributes to the results would improve accessibility for readers less familiar with this approach. [R2] | We have clarified the ZINB inflation component in plain language to improve accessibility:  “*Put simply, the zero-inflation component distinguishes between people who never buy stimulant laxatives, and those who sometimes buy them but had no purchase in a given month. This helps prevent overestimating demand among non-purchasers while still modelling variation among active buyers.*” [line 442] |
| You test several month contrasts (Jan vs Dec; May–Aug vs Sep; interactions by type; multiple categories). Clarify your pre-specified contrasts, control the family-wise error rate (e.g., Holm) or FDR, and present adjusted p-values where appropriate. [R1] | We have clarified in the Methods section which contrasts were pre-specified, and have applied Holm corrections to control the family-wise error rate for the seasonal summer (May–August) vs. September comparisons. Adjusted p-values are now reported in Tables 3-5 as appropriate.  “*For hypotheses involving multiple month contrasts (i.e., H1b, H2b, H3b), we applied Holm corrections to control the family-wise error rate, and report p-values for these adjusted values. All other contrasts were pre-specified single comparisons and therefore did not require multiplicity adjustment.*” [line 461] |
| **Product classification and dosage** |  |
| The stimulant/non-stimulant taxonomy is hand-labelled. Provide a codebook (supplement) listing each SKU, active ingredient(s), assigned class, quantity, and dosage assumptions, plus inter-rater checks if applicable. [R1]  Please expand the methods section (particularly product classification and modelling) for clarity and reproducibility. [R3] | We have provided a full product codebook (active ingredients, stimulant/non-stimulant class, quantity, dosage assumptions) in the Supplementary Material. This is references in the main text:  *“An extended product code book is provided in the Supplementary Material, including the active ingredient in the product, the stimulant classification, and dosage information (with source).”* [line 393] |
| Some items listed as non-stimulant (e.g., guarana, syrup of figs) may be supplements or mixed-purpose—please justify inclusion/exclusion and test robustness, excluding ambiguous products. [R1] | Syrup of figs and guarana were classified according to the retailer’s internal product categorisation, which grouped them under laxatives. We clarify that the data was pre-classified by the retailer (i.e. to laxative level, not to stimulant versus non stimulant):  “*The retailer provided pre-defined product categories, and we used these classifications to identify laxative products from within the larger dataset.*” [line 363]  As such, our classification followed the retailer’s definitions as to what constituted a laxative product, to avoid introducing subjective researcher judgement. Importantly, as neither product contains stimulant laxative ingredients (e.g., senna, bisacodyl), and therefore met our predefined criteria for inclusion in the non-stimulant category. We do acknowledge that some of these products may have mixed purposes or may be used as supplements. However, their presence in the dataset was extremely limited (<3% of all laxative purchases combined). Given this small proportion, their inclusion is unlikely to meaningfully influence our findings.  However, to maintain transparency in the manuscript, we have added clarification on this point:  “*It should be noted that a small number of products have ambiguous classifications (e.g., syrup of figs, guarana), and while included as laxatives under the retailer’s classification system, may also be considered supplements. Yet, as these products did not contain a stimulant ingredient, they were classified within the non-stimulant group according to our pre-defined criteria.”* [line 371] |
| The maximum adult dose assumption could overstate “doses” vs typical use. Add sensitivity analyses using the recommended starting dose and median dose assumptions where available. [R1] | We acknowledge that using the maximum adult dose may overestimate the absolute number of doses relative to typical use. However, our analyses focus on comparative purchasing patterns by season, rather than estimating individual consumption. The maximum-dose assumption provides a consistent metric across buyers, and while using starting or median doses would change absolute values, they are unlikely to affect the direction or relative magnitude of seasonal and type-specific effects, which are the focus of this study.  We have, however, expanded our discussion of this in the limitations:  “*However, this assumption provides a consistent metric across time and buyers, allowing us to reliably examine relative seasonal patterns. Future work could consider alternative dosing assumptions where such data are available.”* [line 293] |
| Weight-management co-purchase analysis:  The reported Pearson correlation (r≈0.11 among overlapping buyers) is weak and ignores time order. [R1] | Following the reviewers suggestion, we used a time-lagged regression to account for time order, looking at purchasing within the same month, and at a 1, 2 and 3 month lag.  This has been reflected in the Methods:  “*To explore temporal associations between weight-management and laxative purchases, we performed time-lagged correlation analyses. We first aggregated purchases by month for the top 10% of weight-management product buyers. Monthly purchase totals for weight-management and laxative products were then aligned, and Pearson correlation coefficients were calculated at lags of 0 to 3 months. Lag 0 represents correlations within the same month, while lag 1–3 represent correlations with weight-management purchases leading laxative purchases by 1–3 months. This approach allowed us to assess whether higher purchases of weight-management products predicted increased laxative purchases in the same or subsequent months.”* [line 465]  And the Results:  *“Time-lagged correlations within this group revealed a mixed temporal relationship. Purchases within the same month (lag 0) were moderately positively correlated (r = 0.39), indicating that individuals who bought more weight-management products in a given month also tended to purchase more laxatives during that month. However, correlations at subsequent months showed alternating directions: 1 month (lag 1) was weakly negative (r = -0.16), 2 months (lag 2) was positive (r = 0.31), and 3 months (lag 3) was negative (r = -0.35). These results suggest that co-purchasing behaviour is not consistent over time and may fluctuate across months.”* [line 180] |
| **Generalisability and Data Limitations** |  |
| Data are from Dec 2013–Dec 2014 and from a single retailer with ~87% female cardholders. Discuss how this affects external validity (gender skew; chain clientele), and whether patterns persist in later years. Please temper any policy-impact language accordingly. [R1]  The dataset is skewed toward female customers and limited to one retailer. Discuss how this affects generalisability. Acknowledge that the data cover 2013–2014 and therefore cannot speak directly to current post-2020 policy impacts. [R3] | We have added the points about female skew/single retailer into our limitations, advising readers to emphasise caution around generalising results:  *“The dataset itself also has representational constraints. It covers transactions from a single UK pharmacy retailer between December 2013 and December 2014, and participants in the dataset are largely female (87%). The gender imbalance likely reflects the retailer’s customer profile rather than national prevalence of laxative use, but caution is necessary in extrapolating patterns to the broader UK population. In addition, the findings may not generalise to modern purchasing environments (>10 years later) or to other retailers and pharmacies.”* [line 300]  Similarly, we clarify that the data predates the 2020 policy change and cannot speak directly to impacts:  *“Likewise, as the data pre-date the 2020 MHRA guidance and the wider expansion of online pharmacies, our results cannot speak directly to current or future impacts. Replication with more recent datasets would help assess whether the observed relationships persist over time and across consumer groups.*” [line 304] |
| **Other** |  |
| The manuscript is generally well-written, though some sentences in the Methods section are long and dense; simplifying these would improve clarity. [R2] | We have simplified the more dense sentences throughout the manuscript. |
| Figures are informative, but y-axis labels could be made more descriptive to enhance interpretability. [R2] | We have adapted the y-axis labels on Figures 1 and 2 to be more descriptive (Mean Number of Products Sold, Mean Number of Doses Purchased). |
| The Data Availability Statement does not currently comply with PLOS policy. While raw data may be restricted by license, you must:   - Provide a clear route for other researchers to request access to the dataset under similar terms. - Deposit all analysis code, product categorisation lists, and dosage conversion lookups in a public repository with a DOI. - Provide de-identified or synthetic datasets (or at least the data underlying summary statistics) to ensure analyses can be reproduced. [R3] | We have updated our Data Availability Statement to comply with PLOS policy, including explaining the route for data requests, depositing analysis code, synthetic data, and product info into a public repository.  *“The data underlying this study consist of pseudonymised supermarket loyalty card transactions provided by a leading UK pharmacy retailer under a data-sharing partnership with the University of Nottingham. Due to legal and commercial confidentiality restrictions, the raw dataset cannot be shared publicly. Researchers interested in accessing the data under equivalent conditions should contact the study team at james.goulding@nottingham.ac.uk, who can provide guidance on the process for applying to the retailer for access.*  *All analysis code, product categorisation lists, dosage conversion scripts, and realistic synthetic data can be accessed via our GitHub repository (https://github.com/RomanaBurgess/loyalty-card-laxatives/).”* [line 492] |
| Please include the ethics approval reference number, details of the retailer data-sharing agreement, and confirmation of GDPR compliance. Clarify what steps were taken to minimise risks of re-identification. [R3]  Please ensure that your Ethics Statement is available in its entirety at the beginning of your Methods section, under a subheading 'Ethics Statement'. | We have moved the ethics statement to the correct area within the methods section, and have clarified the requested details:  *“Approval for this study was granted by the NUBS Research Ethics Review Panel at the University of Nottingham (reference number 201829095). The research used pseudonymised loyalty card transaction data provided under a formal data-sharing agreement and non-disclosure agreement between the University of Nottingham and a major UK pharmacy retailer. The dataset contained no directly identifiable personal information; all data were pseudonymised by the retailer prior to transfer, and no identifiers or linkage keys were available to the research team.*  *The study complied with the UK General Data Protection Regulation (GDPR) and the Data Protection Act 2018. As the data were de-identified with no possibility of re-identifying individuals, informed consent was not required. All analyses were conducted within secure institutional computing environments under the terms of the data governance agreements approved by both the University of Nottingham and the retailer. To completely eliminate any risk of re-identification, outputs are reported only in aggregated form, and no attempt was made to link the loyalty card data to any external sources of personal information.”* [line 332] |
