# Supplementary material for: Using supermarket loyalty card data to investigate seasonal variation in laxative purchases in the UK
Source: PLOS Digit Health. 2026 Mar 18;5(3):e0000963. doi: 10.1371/journal.pdig.0000963 (PMC12998798; doi:10.1371/journal.pdig.0000963)
Supplement: Attachment — Submitted filename: Reviewer Response.docx [file pdig.0000963.s007.docx]

We thank the reviewers for their comments on the manuscript. We have made the changes as suggested, and believe it has led to an improved version of the paper.

1. **In the abstract, please avoid using parentheses for explanations such as “(i.e., painkillers, cold and flu, hay fever, and shampoo)”.**

We have removed brackets from within the abstract.

2. **The manuscript contains a large number of em dashes (“—”). Please remove them.**

We have removed the use of em dashes from the manuscript.

3. **Please include all keywords in the abstract.**

We have added “self-medication” into the abstract: “we analyse **self-medication** purchasing patterns from 748,375 buyers to explore potential links with weight management behaviours.”

4. **Please replace all occurrences of “We” with “This study.”**

We have made the choice to retain usage of “we” throughout the paper, as is consistent with other published articles by the journal (e.g., <https://journals.plos.org/digitalhealth/article?id=10.1371/journal.pdig.0001086>; <https://journals.plos.org/digitalhealth/article?id=10.1371/journal.pdig.0000908>).

5. **The reference list is too limited. Please add more recent references from the past few years.**

We have added a handful of more recent references where appropriate.

- Dawson SW, Le DQ, Tan EJ, Le LKD. The regulation on the use of supplements for weight control: Case studies from Australia, the United States of America, and the United Kingdom. Health Policy OPEN. 2025 Jun 1;8:100136.
- MacNeil BA, Gorman T, Maier J. Examining the associations between laxative use, substance use, depressive symptoms, and obsessions and compulsions in adults with an eating disorder. Journal of Psychiatric Research. 2025 Feb 1;182:142–8. doi:10.1016/j.jpsychires.2024.12.043
- Patil K, Taskar A, Benke S, Ahire K, Chaudhari B, Bairagi V. Misuse and Abuse of Over the Counter Medication: A Growing Public Health Concern. International Journal of Scientific Research and Technology. 2025 Dec 19. Located at: World. doi:10.5281/zenodo.17991170
- Burgess R, Suhag A, Skatova A. Exploring the use of supermarket loyalty card data in health research: A scoping review. Public Health. 2025 Oct 1;247:105848. doi:10.1016/j.puhe.2025.105848
- Skatova A. Overcoming biases of individual level shopping history data in health research. npj Digit Med. 2024 Sep 30;7(1):1–5. doi:10.1038/s41746-024-01231-4

6. **Please add a study workflow flowchart and provide an explanation of the research process.**

We have added a Figure (Fig 3) to showcase the research process, including pre-processing, analysis and interpretation stages. This includes the initial and analytic sample sizes. However, we have added no further explanation of the research process as we feel this is covered throughout the methods and do not wish to lengthen the word count where possible.
